# Transient Changes in Brain Metabolites after Transcranial Direct Current Stimulation in Spastic Cerebral Palsy: A Pilot Study

**DOI:** 10.3389/fneur.2017.00366

**Published:** 2017-07-31

**Authors:** Paradee Auvichayapat, Benchaporn Aree-uea, Narong Auvichayapat, Warinthorn Phuttharak, Taweesak Janyacharoen, Orathai Tunkamnerdthai, Wuttisak Boonphongsathian, Niran Ngernyam, Keattichai Keeratitanont

**Affiliations:** ^1^Department of Physiology, Faculty of Medicine, Khon Kaen University, Khon Kaen, Thailand; ^2^Division of Pediatric Neurology, Department of Pediatrics, Faculty of Medicine, Khon Kaen University, Khon Kaen, Thailand; ^3^Department of Radiology, Faculty of Medicine, Khon Kaen University, Khon Kaen, Thailand; ^4^Department of Physical Therapy, Faculty of Associated Medical Sciences, Khon Kaen University, Khon Kaen, Thailand

**Keywords:** magnetic resonance spectroscopy, brain metabolites, basal ganglia, primary motor cortex, transcranial direct current stimulation, spastic cerebral palsy

## Abstract

**Background:**

Muscle spasticity is a disability caused by damage to the pyramidal system. Standard treatments for spasticity include muscle stretching, antispastic medications, and tendon release surgeries, but treatment outcomes remain unsatisfactory. Anodal transcranial direct current stimulation (tDCS) in patients with muscle spasticity is known to result in significant improvement in spastic tone (*p* < 0.001). However, the mechanism of action by which tDCS treatment affects spasticity remains unclear. This pilot study aimed to investigate the effect of anodal tDCS upon brain metabolites in the left basal ganglia and ipsilateral primary motor cortex (M1) in children with spastic cerebral palsy (CP).

**Materials and methods:**

This study consisted of three steps: a baseline evaluation, a treatment period, and a follow-up period. During the treatment period, patients were given 20 min of 1 mA anodal tDCS over the left M1 for five consecutive days. Outcomes were compared between pre- and immediate posttreatment in terms of brain metabolites, Tardieu scales, and the quality of upper extremity skills test.

**Results:**

Ten patients with spastic CP were enrolled. Following tDCS, there were significant increases in the ratio of N-acetylaspartate (NAA)/creatine (Cr) (*p* = 0.030), choline (Cho)/Cr (*p* = 0.043), and myoinositol (mI)/Cr (*p* = 0.035) in the basal ganglia. Moreover, increased glutamine–glutamate (Glx)/Cr ratio in the left M1 (*p* = 0.008) was found. In addition, we also observed improvements in the extent of spasticity and hand function (*p* = 0.028).

**Conclusion:**

Five consecutive days of anodal tDCS over the left M1 appeared statistically to reduce the degree of spasticity and increase NAA, Cho, mI, and Glx. Future research studies, involving a larger sample size of spastic CP patients undergoing tDCS is now warranted.

## Introduction

Muscle spasticity is a sign of an upper motor neuron deficit caused by damage to the pyramidal system; this reduces cortical input to the corticospinal tract, resulting in the disinhibition of spinal segmental excitability and an increase in muscle tone (1). Standard treatments for spasticity include muscle stretching, antispastic medications, and tendon release surgeries (1). However, no treatment has yet been found, which successfully eliminates spasticity in all individuals (1).

Previous research studies have demonstrated the effects of anodal transcranial direct current stimulation (tDCS) upon motor function of the upper (2) and lower limbs (3–5) in patients with spastic cerebral palsy (CP). However, the assessment of spasticity or motor function in the upper limbs is preferable because defects in arm-hand performance due to stroke or CP lead to greater problems in daily life than defects involving the lower limbs (6–8).

Grecco et al. determined the effect of tDCS applied over the primary motor cortex (M1) for 20 min during treadmill training on the gait pattern of children with spastic diparetic CP. The experimental group showed improvements in both temporal functional mobility and gait variables. The researchers suggested that tDCS during treadmill training potentiated the effects of motor training in children with spastic diparetic CP (3). Moreover, gait training on a treadmill, combined with anodal stimulation of the M1, has led to improvements in static balance and functional performance in children with CP (4). It has also been reported that anodal tDCS induces changes in the excitability of the motor cortex controlling the lower limbs, with improvements in both balance and gait (5). Our previous study found that anodal tDCS can reduce upper limb spasticity and increase the range of motion in children with spastic CP (2). These earlier results illustrated statistically significant differences in Ashworth scores between groups after immediate posttreatment, and this improvement in spasticity was maintained for at least 48 h (2). Therefore, anodal tDCS applied to M1 in children with CP can momentarily potentiate motor patterns through the enhancement of cortex excitability and the activation of corticospinal excitability. Furthermore, the facilitation of cortical excitability of the M1 may enhance motor control in children with CP.

In spasticity arising from stroke, Ochi et al. demonstrated the effects of 1 mA of anodal tDCS over the affected hemisphere for 5 days in chronic stroke patients; results revealed significant improvements in spasticity and functional arm movement measured using the Fugl-Meyer Assessment for the upper limbs (9). In addition, Viana et al. reported improvement of wrist spasticity in their anodal tDCS group (10). Collectively, these results suggest that anodal tDCS over the motor cortex may exert a beneficial role in reducing the degree of spasticity. However, the precise mechanism of action underlying the effects of tDCS treatment upon spasticity remains unclear.

Proton magnetic resonance spectroscopy (1H-MRS) is a non-invasive diagnostic test for measuring biochemical changes in the brain (11). 1H-MRS studies of the brain are capable of quantifying steady-state metabolic levels of neurotransmitters, such as N-acetylaspartate (NAA), a marker for neuronal and axonal integrity. Several small molecules form major constituents of the cell membrane and represent useful markers for membrane turnover and reflect neuronal connections (12); one such example is choline (Cho). In addition, glutamine–glutamate (Glx) is an important excitatory neurotransmitter, while myoinositol (mI) is an osmolyte and astrocyte marker, which reflects synaptogenesis (13, 14). Finally, creatine (Cr) is often used as an internal standard to which the resonance intensities of other metabolites are normalized (12).

In a previous study, 1H-MRS was performed in the basal ganglia of children with dyskinetic and spastic CP; analysis revealed reductions in NAA/Cr and Cho/Cr (15). Kulak et al. also reported reductions of both NAA and Cho in the basal ganglia (16). Another study reported that children with spastic CP showed reduced NAA/Cr, NAA/Cho, NAA/mI, and GABA/Cr relative to a control group (17). Moreover, an age-dependent increase of NAA was observed in healthy children but not in children with CP (18).

Some studies, involving 1H-MRS, have demonstrated that anodal tDCS is associated with an increase of various neurochemicals. For example, Clark et al. reported the detection of increased levels of both Glx and mI beneath the stimulating electrode (19), while Rango et al. reported a significant increase in mI, but neither NAA, nor other important metabolites, showed any change following anodal or sham stimulation (20). Conversely, Stagg et al. reported a reduction of GABA concentration following anodal stimulation tDCS (21). Changes in glutamatergic and GABAergic activity may translate to subsequent alterations in both local and distributed processing, influenced by both excitatory and inhibitory signaling pathways in functional brain networks (22). As mentioned above, anodal tDCS can reduce spasticity, as evaluated by clinical assessment alone (2, 9). Therefore, the present study aimed to investigate the effect of left M1 anodal tDCS upon brain metabolites in the left basal ganglia and ipsilateral M1 in children with spastic CP.

## Materials and Methods

### Participant Recruitment and Informed Consent

This pilot study was an experimental study aiming to compare the efficacy of tDCS by carrying out assessments before and after treatment (pre–post study design). Spastic CP was defined according to the standard criteria for CP diagnosis in children over 1 year of age (23). The abilities and limitations of the recruited CP patients, in terms of gross motor function, were defined using the Gross Motor Functional Classification System (24). Clinical spasticity was diagnosed according to Tardieu scales (TS) (25). Participants were recruited by an advertisement in the Pediatric Outpatient Neurology Department, Srinagarind Hospital, Khon Kaen, Thailand. The inclusion criteria were as follows: diagnosis of spastic CP according to standard criteria for CP diagnosis (23); Gross Motor Functional Classification System Level I–II (24); boys with spastic CP aged 8–12 years; and right upper limb spasticity grades 1–3 according to the TS (25). Patients were excluded if they met any of the following criteria: presence of a pacemaker; drug abuse; non-cooperative patients and caregivers; history of craniotomy or skull defects; severe neurological disorders, such as brain tumors, intracranial infection, or intellectual disabilities; uncontrollable epilepsy, defined as the occurrence of seizures despite the use of at least one antiepileptic drug at an adequate dose; subjects who received other treatments, such as herbal medicine, massage, or an intrathecal baclofen pump; orthopedic surgery on the upper limbs less than 6 months before enrollment; initiation or dose change of oral antispastic medication within 5 days of the study; or Botulinum toxin (type A or B) injections less than 90 days before enrollment.

Ten subjects participated in this study; the need for this sample size was determined according to the number required for appropriate statistical power in a pilot study. All participants provided verbal informed consent to participate. In addition, the guardians of all participants provided written informed consent. This study conformed to the Declaration of Helsinki and was approved by the Ethics Committee at Khon Kaen University (identifier number HE 561111).

### Experimental Design

The study protocol consisted of three steps: baseline evaluation, a treatment period, and an immediate follow-up period. This allowed comparison of the outcomes between pre- and posttreatment. During the baseline evaluation step, we collected a dataset for each subject, including MRS, TS, and the quality of upper extremity skills test (QUEST). During the treatment step, 10 children with spastic CP underwent 20 min of 1 mA anodal tDCS stimulation over the left M1 for five consecutive days. Finally, during the follow-up step, subjects underwent MRS, the TS, and QUEST immediately after the final tDCS stimulation. Then, outcomes were compared between pre- and posttreatment.

#### Blinding and Kappa Reporting

Staff members who recruited participants were not involved in any of the subsequent assessments. After the intervention, the neuroradiologist who carried out the brain metabolite assessments (WP) was blinded to both pre- and posttreatment conditions.

The TS and QUEST were performed upon entry, and the end of the treatment, by two trained professional physical therapists (Taweesak Janyacharoen and Benchaporn Aree-uea) who were not involved in the research treatment and were also blinded to pre- and posttreatment status. These therapists came from two centers and were trained to evaluate footage of spastic CP patients. Inter-therapist reliability was assessed by κ statistics and was demonstrated to be of a high level (Cohen’s κ = 0.905; 95% confidence interval = 0.850–0.959).

### Assessments

#### Magnetic Resonance Spectroscopy

**Figure 1:**
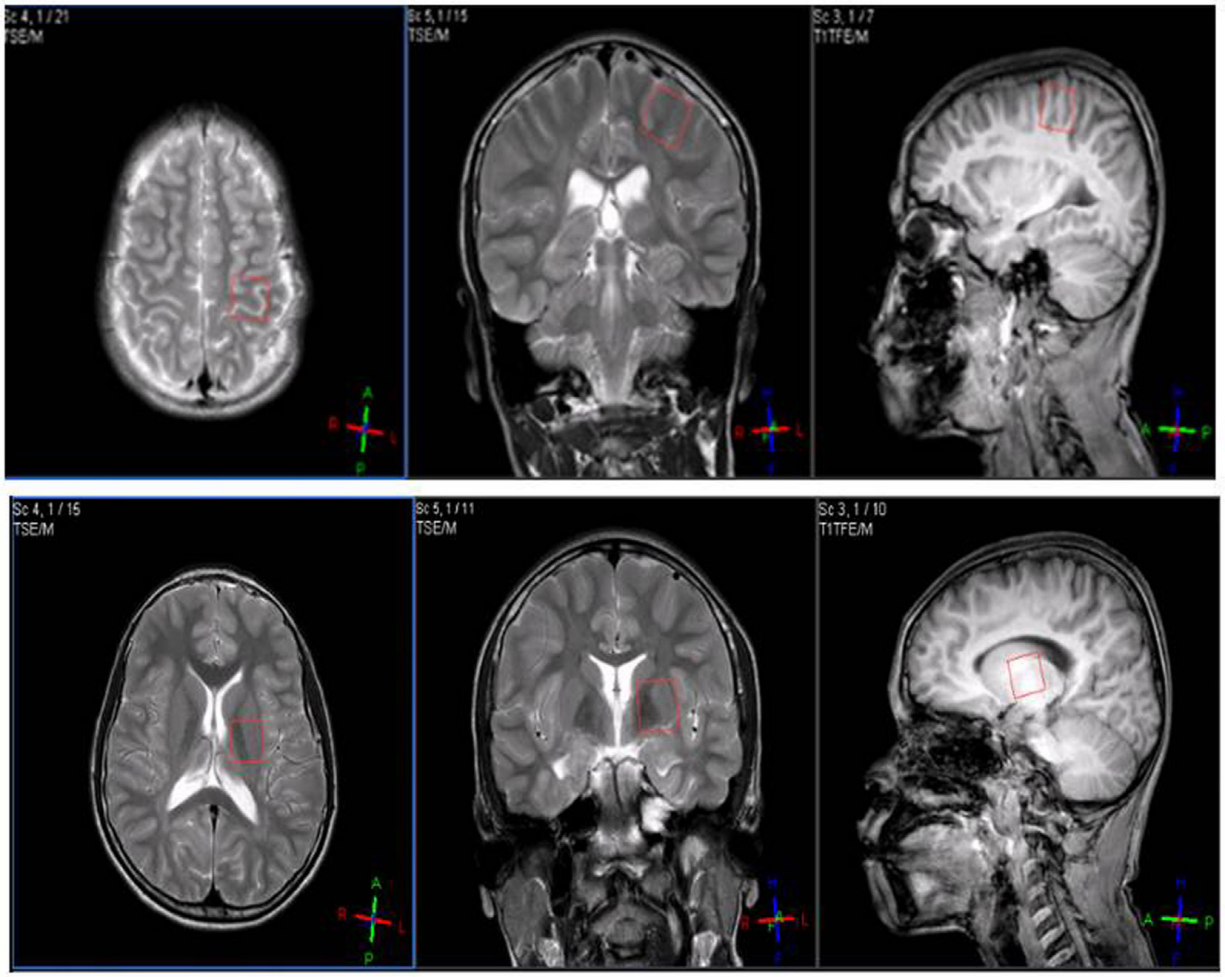
Single voxels (2 cm × 2 cm × 2 cm) were positioned on coronal, sagittal, and axial images from the areas of the motor cortex (top) and basal ganglia (down).

The subjects participated in MRS sessions before and after the final tDCS stimulation in the Diagnosis Radiology Unit, Department of Radiology, Faculty of Medicine. We performed 1H-MRS using a Philips Achieva 3.0T (Philips Healthcare, Best, Netherlands) running 2.6.3.3 MR workspace software. The subjects were instructed to lie still for approximately 40 min for data collection. Single-voxel 1H-MR spectra were then acquired and quantified with an LC Model (Stephen Provencher Inc., Oakville, ON, Canada) to determine metabolite concentration ratios. Single voxels (2 cm × 2 cm × 2 cm) were positioned on coronal, sagittal, and axial images from the areas of the left motor cortex and basal ganglia (Figure 1). Spectra were acquired using a point resolved spectroscopy (PRESS) sequence with a TR of 2 s, short TE of 35 ms, spectral width of 2,000 Hz, 1,024 time points, and partial water suppression. Shimming was performed using shimming procedures provided by the manufacturer. Analysis of metabolite concentrations was carried out using an LC Model. Levels of NAA, Cr, Cho, mI, and Glx were analyzed by fitting a linear combination of a basis set of metabolite model spectra to the data. The analyzing spectrum was set from 3.8 ppm down to 0.2 ppm without eddy-current correction or water scaling. All metabolite concentrations were expressed as millimoles. The relative concentrations of NAA, Cho, mI, and Glx were measured relative to Cr and different combinations of metabolites within 8 cm^3^ brain voxels. The MRS session was controlled by an expert radiological technologist (Wuttisak Boonphongsathian).

#### Tardieu Scale

The TS was measured by passive movement of the joints at three specified velocities: slow, under gravity, and fast; these are referred to hereafter as V1, V2, and V3, respectively. The TS was used to assess muscle tone around the shoulder, elbow, and wrist and finger joints on the right side, opposite to the stimulated M1. Grading was performed regularly at the same time every day, in a constant position of the body on the right upper limb. Responses were recorded at each velocity as *X*/*Y*, where *X* was the intensity of the muscle reaction to stretching indicated by a rating of 0–5 (0 = no resistance throughout the course of passive movement, 5 = joint was immovable), and *Y* indicated the angle (in degrees) at which the muscle reaction occurred (25, 26).

#### The Hand Function Test

Quality of upper extremity skills test was used to measure both the movement and function of the left and right upper limbs in each subject. The present study evaluated the quality of upper extremity function in three domains: dissociated movement, grasp, and weight bearing (27), and the total score was thus determined.

#### Transcranial Direct Current Stimulation

Direct current was transferred using a saline-soaked pair of surface sponge electrodes (35 cm^2^) and delivered by a battery-driven power supply. Constant current stimulation, with a maximum output of 10 mA, was delivered by a Soterix Medical System, Model 1224-B (New York, NY, USA). The anodal electrode was placed over the left M1 and the cathodal electrode was placed over the contralateral shoulder area. A constant current of an intensity of 1 mA and duration of 20 min was applied for five consecutive days.

### Statistical Analysis

Analyses were performed using Stata software, version 10.0 (StataCorp, College Station, TX, USA). The primary outcome was the determination of brain metabolite concentrations, while the secondary outcome was the evaluation of spasticity and hand function. A paired *t*-test was used if data were normally distributed, and the results were reported as mean differences with 95% confidence intervals. In contrast, if a non-normal distribution was identified, we used the Wilcoxon signed-rank test and reported median differences with 95% confidence intervals. A *p*-value less than 0.05 was considered to be statistically significant.

## Results

A total of 12 right upper limb spastic CP patients were enrolled between November 2011 and February 2013. Ten of these subjects met with our specific inclusion criteria. Two participants were excluded because they did not complete the study; one dropped out on the third day of the treatment period, while the other dropped out at the baseline brain metabolite assessment stage. Consequently, MRI was performed in 10 children with spastic CP. A summary of demographic data, along with the clinical characteristics of each subject, are presented in Table 1.

**Table 1:** The demographic data and characteristics of the subjects (*n* = 10).

Age (years) (mean ± SD)	10.4 ± 1.65
Age at diagnosis (years old)	
0–1	2
1–2	5
2–3	3
Spasticity subtype	
Spastic diplegia	3
Right spastic hemiplegia	6
Spastic quadriplegia (bilateral hemiplegia)	1
Delivery	
Term	8
Preterm	2
Birth weight (g) (mean ± SD)	2,822 ± 942
Etiologies
– Congenital brain anomalies	4
• Periventricular leukomalacia	2
• Pachygyria	1
• Schizencephaly	1
– Birth trauma	2
• Subdural hemorrhage	2
– Prematurity	2
• Intraventricular hemorrhage	2
– Idiopathic	2
Quality of upper extremity skills test (The QUEST)	
Dissociated movement	71.43 ± 22.83
Grasps	69.65 ± 21.31
Weight bearing	64.10 ± 25.98
Total score	68.26 ± 22.23

### Metabolite Concentration Changes

There was a significant increase in the concentrations of several brain metabolites (NAA, Cho, and mI) in the left basal ganglia (*p* = 0.013, 0.007, and 0.020, respectively). However, there were no significant differences in the concentrations of Glx or Cr between pre- and posttreatment (*p* = 0.355, and 0.374, respectively) (Table 2).

**Table 2:** Metabolite concentrations in left motor cortex and left basal ganglia between pre- and posttreatment.

Metabolites (mM)	Motor cortex			Basal ganglia		
	Pretreatment (*n* = 10)	Posttreatment (*n* = 10)	*p*-Value	Pretreatment (*n* = 10)	Posttreatment (*n* = 10)	*p*-Value
NAA	2.04 ± 0.25	2.06 ± 0.28	0.588^a^	1.38 ± 0.31	1.58 ± 0.18	0.013^b^*
Cho	0.76 ± 0.11	0.75 ± 0.09	0.509^a^	0.64 ± 0.18	0.73 ± 0.13	0.007^a^*
mI	0.48 ± 0.07	0.48 ± 0.08	0.667^a^	0.36 ± 0.11	0.43 ± 0.06	0.020^a^*
Glx	0.32 ± 0.04	0.34 ± 0.05	0.070^a^	0.22 ± 0.06	0.23 ± 0.04	0.355^a^
Cr	1.23 ± 0.14	1.22 ± 0.14	0.838^b^	1.12 ± 0.16	1.16 ± 0.11	0.374^b^

*Values are means ± SD*.

*Represent the significant differences from baseline (*p* < 0.05).

*^a^Data were analyzed by paired t-test*.

*^b^Data were analyzed by Wilcoxon signed ranks test*.

*NAA: N-acetylaspartate; Cho: Choline; mI: myoinositol; Glx: Glutamine and glutamate; Cr: Creatine*.

### Metabolite Ratio Changes

Our results showed a significant increase in Glx/Cr ratio in the left M1 (*p* = 0.008). The NAA/Cr, Cho/Cr, and mI/Cr ratios in the left basal ganglia were also increased after tDCS treatment (*p* = 0.030, 0.043, and 0.035, respectively) (Table 3).

**Table 3:** Metabolite ratios in left motor cortex and left basal ganglia between pre- and posttreatment.

Metabolites	Motor cortex			Basal ganglia		
	Pretreatment (*n* = 10)	Posttreatment (*n* = 10)	*p*-Value	Pretreatment (*n* = 10)	Posttreatment (*n* = 10)	*p*-Value
NAA/Cr	1.66 ± 0.09	1.69 ± 0.13	0.145^a^	1.24 ± 0.20	1.37 ± 0.16	0.030^a^*
NAA/Cho	2.70 ± 0.26	2.77 ± 0.22	0.169^b^	2.19 ± 0.24	2.19 ± 0.30	0.974^a^
NAA/mI	4.27 ± 0.35	4.30 ± 0.51	0.959^b^	3.88 ± 1.07	3.76 ± 0.66	0.674^a^
Cho/Cr	0.62 ± 0.05	0.61 ± 0.02	0.905^a^	0.57 ± 0.11	0.63 ± 0.08	0.043^a^*
Cho/NAA	0.37 ± 0.03	0.36 ± 0.03	0.386^b^	0.46 ± 0.06	0.46 ± 0.06	0.961^a^
Cho/mI	1.58 ± 0.10	1.56 ± 0.10	0.612^a^	1.90 ± 0.50	1.75 ± 0.35	0.203^b^
mI/Cr	0.39 ± 0.02	0.40 ± 0.04	0.357^a^	0.32 ± 0.09	0.37 ± 0.06	0.035^a^*
mI/NAA	0.24 ± 0.02	0.23 ± 0.02	0.911^a^	0.26 ± 0.09	0.27 ± 0.04	0.508^b^
mI/Cho	0.63 ± 0.04	0.64 ± 0.04	0.597^a^	0.58 ± 0.23	0.60 ± 0.14	0.386^b^
Glx/Cr	0.26 ± 0.02	0.28 ± 0.03	0.008^a^*	0.19 ± 0.03	0.20 ± 0.03	0.618^a^

*Values are means ± SD*.

*Represent the significant differences from baseline (*p* < 0.05).

*^a^Data were analyzed by paired t-test*.

*^b^Data were analyzed by Wilcoxon signed ranks test. NAA, N-acetylaspartate; Cho, Choline; ml, myoinositol; Glx, Glutamine and glutamate; Cr, Creatine*.

### Tardieu Scale (Spasticity Grade and Angle)

The Wilcoxon signed-rank test revealed a significant improvement in the spasticity grade (TS) for the right shoulder flexors, shoulder adductors, shoulder internal rotators, shoulder external rotators, elbow extensors, and wrist flexors compared to baseline (*p* = 0.046, 0.014, 0.025, 0.034, 0.007, and 0.025, respectively). Analysis also showed a significant reduction in Tardieu spasticity angles (Xv1–Xv3) in the right shoulder extensors, shoulder adductors, shoulder abductors, elbow extensors, and elbow pronators (*p* = 0.043, 0.034, 0.041, 0.012, and 0.026, respectively) (Table 4).

**Table 4:** Tardieu scale (TS) of patients at pre- and post-tDCS treatment (*n* = 10).

Clinical measures	Pretreatment	Posttreatment	*p*-Value
TS															
**Shoulder flexors**			
Tardieu score	0.80 (1) (0.79)	0.40 (0) (0.52)	0.046*
Tardieu spasticity angle	7.50 (5) (8.25)	6.00 (0) (8.76)	0.673
**Shoulder extensors**			
Tardieu score	0.60 (0.5) (0.70)	0.30 (0) (0.48)	0.257
Tardieu spasticity angle	25.00 (15) (28.09)	4.00 (0) (8.76)	0.043*
**Shoulder adductors**			
Tardieu score	0.80 (1) (0.63)	0.20 (0) (0.42)	0.014*
Tardieu spasticity angle	41.30 (41.5) (35.00)	15.00 (0) (38.08)	0.034
**Shoulder abductors**			
Tardieu score	0.60 (0.5) (0.70)	0.10 (0) (0.32)	0.059
Tardieu spasticity angle	8.00 (10) (7.53)	1.00 (0) (3.16)	0.041*
**Shoulder internal rotators**			
Tardieu score	0.70 (0.5) (0.82)	0.20 (0) (0.42)	0.025*
Tardieu spasticity angle	20.50 (15) (22.91)	7.50 (0) (16.87)	0.104
**Shoulder external rotators**			
Tardieu score	1.10 (1) (0.88)	0.50 (0) (0.71)	0.034*
Tardieu spasticity angle	24.00 (20) (22.09)	9.50 (0) (15.17)	0.061
**Elbow flexors**			
Tardieu score	1.40 (1.5) (0.70)	1.00 (1) (0.67)	0.102
Tardieu spasticity angle	65.00 (75) (33.00)	76.50 (90) (30.00)	0.514
**Elbow extensors**			
Tardieu score	1.10 (1) (0.74)	0.20 (0) (0.42)	0.007*
Tardieu spasticity angle	32.20 (22.5) (29.30)	5.50 (0) (11.65)	0.012*
**Elbow supinators**			
Tardieu score	0.20 (0) (0.42)	0.10 (0) (0.32)	0.317
Tardieu spasticity angle	1.50 (0) (3.37)	0.50 (0) (1.58)	0.317
**Elbow pronators**			
Tardieu score	0.60 (1) (0.52)	0.50 (0.5) (0.53)	0.317
Tardieu spasticity angle	19.50 (15) (20.74)	9.00 (5) (10.22)	0.026*
**Wrist flexors**			
Tardieu score	0.80 (1) 0.63	0.30 (0) 0.67	0.025*
Tardieu spasticity angle	20.50 (15) 20.47	6.50 (0) 18.86	0.075
**Wrist extensors**			
Tardieu score	0.30 (0) 0.48	0.10 (0) 0.32	0.157
Tardieu spasticity angle	4.00 (2) 6.99	1.70 (2) 5.38	0.285
**Wrist ulnar deviators**			
Tardieu score	0.20 (0) 0.42	0.10 (0) 0.32	0.317
Tardieu spasticity angle	1.50 (0) 3.17	0.60 (0) 1.90	0.180
**Wrist radial deviators**			
Tardieu score	0.20 (0) 0.42	0.10 (0) 0.32	0.317
Tardieu spasticity angle	1.40 (0) 3.10	0.50 (0) 1.58	0.317
**Finger flexors**			
Tardieu score	0.30 (0) 0.48	0.10 (0) 0.32	0.157
Tardieu spasticity angle	5.50 (0) 10.12	0.50 (0) 1.58	0.109

*Values are means (median) (SD)*.

*Represent the significant differences from baseline (*p* < 0.05) by Wilcoxon signed ranks test.

### Correlation between Metabolite Ratios and the Tardieu Scale

**Figure 2:**
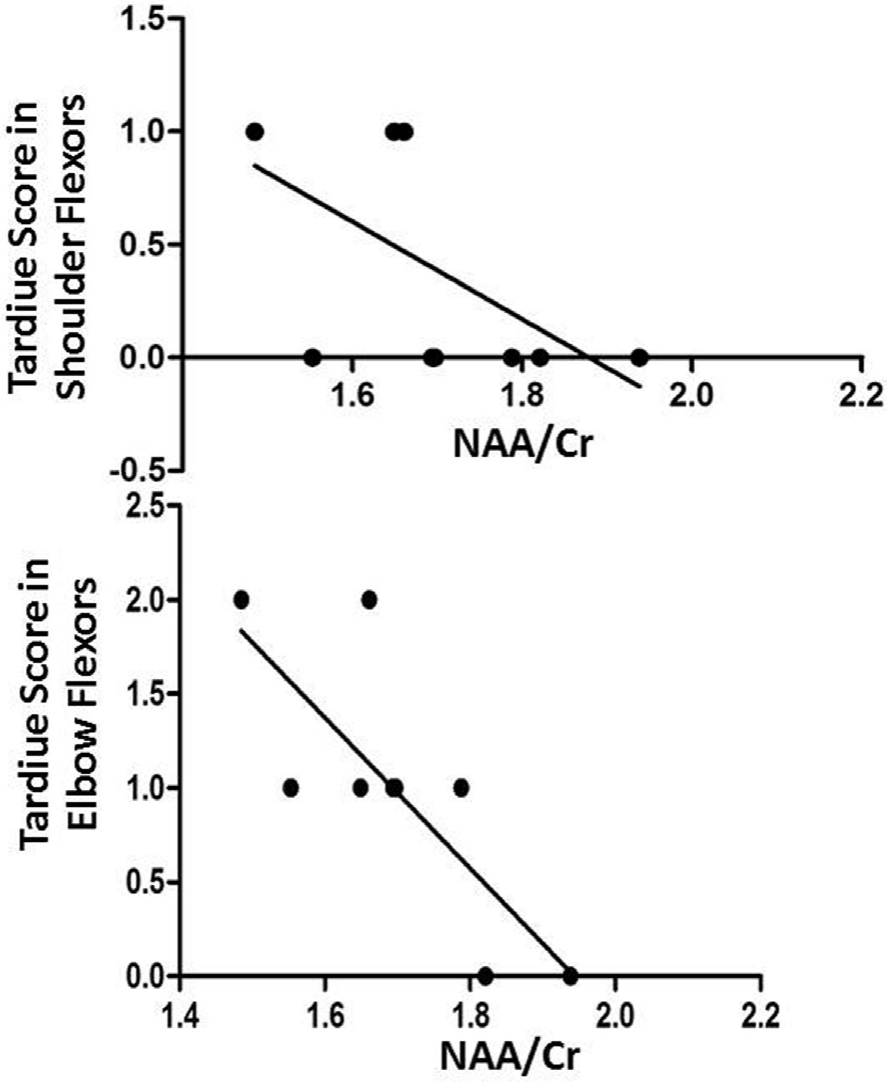
Negative correlations after transcranial direct current stimulation between NAA/Cr in the left basal ganglia and Tardiue score in the right shoulder flexors (*r* = −0.642; *p* = 0.046) and right elbow flexors (*r* = −0.733; *p* = 0.016).

We calculated the Spearman’s rank correlation coefficient to determine the correlation between metabolite ratios and the TS. Our analysis revealed significant negative correlations after tDCS between NAA/Cr in the left basal ganglia and the TS in the right shoulder flexors (*r* = −0.642; *p* = 0.046) and right elbow flexors (*r* = −0.733; *p* = 0.016) (Figure 2).

**Figure 3:**
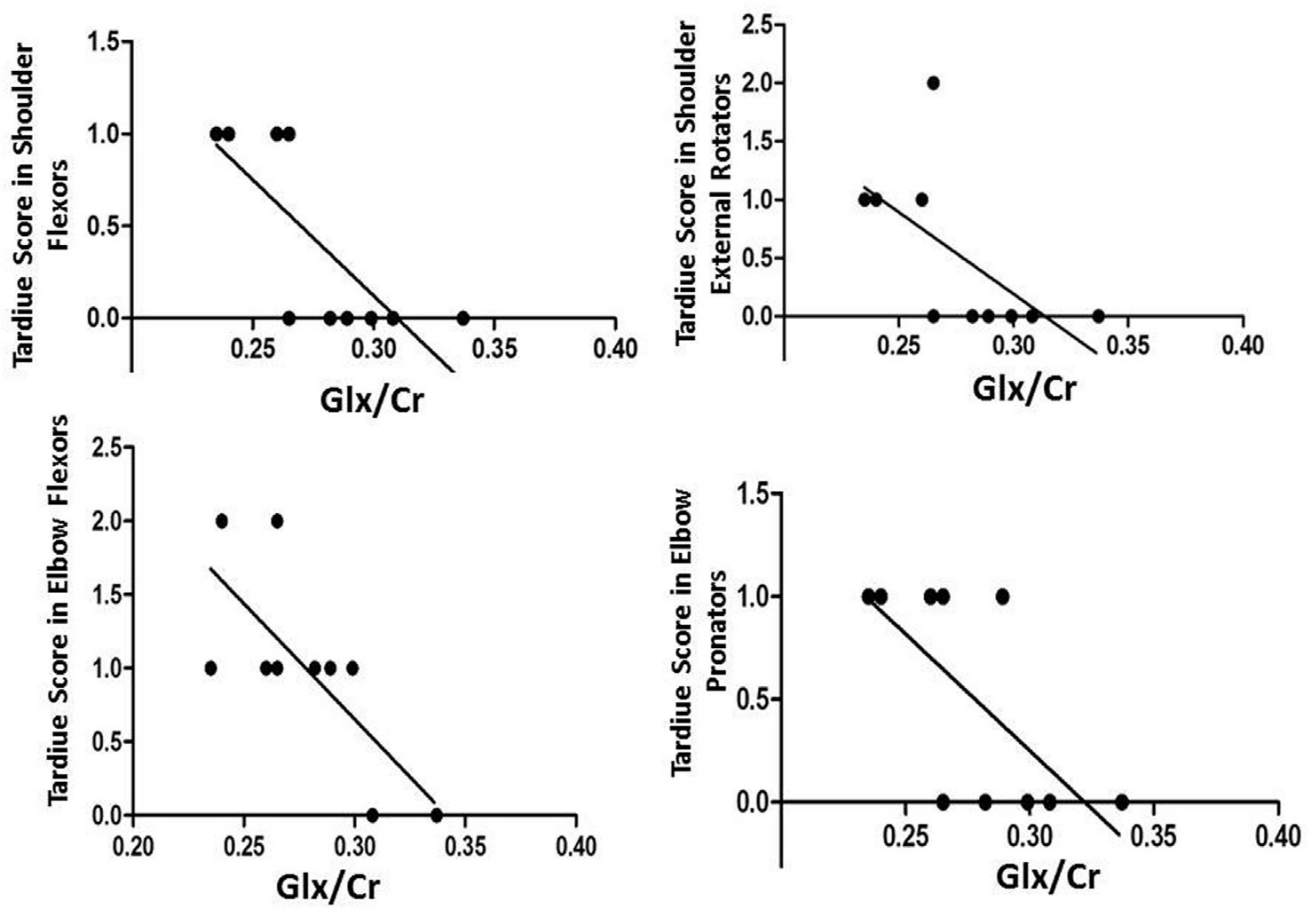
Negative correlations after transcranial direct current stimulation between Glx/Cr in the left primary motor cortex and Tardiue score in the right shoulder flexors (*r* = −0.820; *p* = 0.004), right shoulder external rotators (*r* = −0.748; *p* = 0.013), right elbow flexors (*r* = −0.690; *p* = 0.027), and right elbow pronators (*r* = −0.698; *p* = 0.025).

We also found significant negative correlations after tDCS between Glx/Cr in the left M1 and TS in the right shoulder flexors (*r* = −0.820; *p* = 0.004), right shoulder external rotators (*r* = −0.748; *p* = 0.013), right elbow flexors (*r* = −0.690; *p* = 0.027), and right elbow pronators (*r* = −0.698; *p* = 0.025) (Figure 3).

### The Quality of Upper Extremity Skills Test

Total QUEST scores revealed a statistically significant improvement (*p* = 0.028) when compared between pre- and posttreatment. With respect to each item, there was a significant improvement in dissociated movement (*p* = 0.028), but not for grasp (*p* = 0.113) or weight bearing (*p* = 0.343) when compared between pre- and posttreatment.

## Discussion

To the best of our knowledge, this is the first study to examine brain metabolite changes after anodal tDCS over the left M1 in children with spastic CP. The primary outcome revealed a significant increase in the ratios of NAA/Cr, Cho/Cr, and mI/Cr in the left basal ganglia and Glx/Cr in the left motor cortex after treatment. We also found a negative correlation after treatment between NAA/Cr, Glx/Cr, and the TS. In addition, we also observed improvements in the degree of spasticity and hand function.

Because this is the first study to demonstrate the effect of anodal tDCS in patients with spastic CP by MRS, a direct comparison with previously published results is not possible. However, our study showed positive effects of anodal tDCS due to increased levels of NAA in the left basal ganglia. NAA is a marker of neuronal function involved in synaptic processes and can be considered as a neuronal and axonal marker (12). The increase in NAA observed in the basal ganglia after tDCS in the present study is concordant with studies involving healthy subjects (19, 28). In addition, previous studies involving spastic CP patients reported reductions of NAA (16), NAA/Cr (15), NAA/Cho, and NAA/mI (16). The fact that we observed increased NAA/Cr ratios can support the claim that anodal tDCS is likely to reflect the upregulation of neuronal activity modulation or increased synaptic density in the basal ganglia of spastic CP patients.

Our findings also showed increased concentrations of Cho and mI in the basal ganglia. Previous studies reported lower concentrations of Cho and mI in patients with spastic CP compared to a normal group (15–17). Cho is the precursor for phosphatidylcholine and represents a marker of cellular density as it forms a major constituent of the cell membrane. Moreover, this choline-containing compound is a precursor for the biosynthesis of acetylcholine (12, 29), a neurotransmitter used in large aspiny neurons, which are a specific type of interneuron found in the striatum (30). Therefore, increasing concentrations of Cho may impact upon neuronal connections. In the brain, mI is derived from the recycling of inositol-containing phospholipids, which are linked to membrane phospholipids and their metabolism (31, 32). Anodal tDCS may, therefore, be able to influence membrane phospholipid metabolism in the basal ganglia, change the mI concentration (20), and play a role in the synthesis of inositol-containing phospholipids during synaptogenesis, axonal growth, and myelination (12, 14).

Our other important outcome revealed a significant increase in Glx/Cr beneath the site of tDCS stimulation, as also reported in a previous study (19). Glutamate is the major excitatory neurotransmitter in the brain and the metabolism and transport of this molecule is responsible for the largest neurotransmitter-related energy utilization in the brain (33). Alteration in the concentrations of glutamate and glutamine may indicate changes in glutamatergic neurotransmission, thus resulting in neural activation and increased metabolism (19).

Basal ganglia are strongly interconnected and receive their primary input from the motor cortex (30). In muscle spasticity resulting from motor cortex damage, impulse transmission in the immediately adjacent descending cortical tract is interrupted. The consequence of this is reduced activity, not only of the pyramidal tract but also other areas, including the basal ganglia (1). Our study revealed an increase in Glx in response to anodal tDCS on the M1. This result may indicate that tDCS on the M1 can modify the function of the basal ganglia, reflected by the increased concentrations of NAA, Cho, and mI in the basal ganglia.

It is interesting that direct stimulation of the motor cortex resulted in a more significant change in some metabolites in the basal ganglia than in the motor cortex. Previous studies have shown that baseline concentrations of neurometabolites in the basal ganglia of children with CP are lower than they are in normal children of the same age (15–18), while NAA/Cr ratios in the motor cortex are higher in patients with spastic CP than they are in normal children (34). These results suggest that higher baseline concentrations of brain metabolites in the motor cortex have a lower opportunity to increase further; in contrast, lower basal metabolites in the basal ganglia have greater opportunity to increase further. The links between the motor cortex and basal ganglia are complex and are described in more detail above.

## Summary and Limitations

This is the first study to describe the changes in brain metabolite concentrations, which occur after 1 mA, 20-min anodal tDCS over the M1 for five consecutive days in children with spastic CP. These findings support previous studies showing the potential benefits of tDCS for children with spastic CP. The present data suggest that these benefits may relate to an increase in some brain metabolites in the left M1 region and basal ganglia. However, this study has some limitations, which need to be taken into consideration. First, our study did not seek to treat or assess spasticity or motor function of the lower limbs because the motor homunculus of the leg area is located deep in the interhemispheric fissure, which may be too far away from tDCS currents on the scalp. Second, the measurement parameters (brain metabolite changes) were performed immediately after tDCS stimulation and not at any other timepoint. Thus, we were not able to evaluate the long-term effects of stimulation. Third, we should report changes of brain metabolites according to laterality, whether it affects the contralateral brain or not. However, studying several different areas of the brain would be very time consuming and it is very difficult for children with spastic CP to remain stationary in the scanner for long periods of time. Finally, this study was performed on 10 children with spastic CP. This appears to be a rather small sample size; however, this represented 37–59% of the sample size of a previous full-scale study of human MRS on CP patients (15–18). This indicates that our current sample size was sufficiently accurate at the statistical level to meet a variety of possible aims (35). In addition, the subjects involved in this study were also vulnerable; therefore, it was appropriate to use the smallest possible sample size that could answer the research question for this particularly vulnerable group.

## Ethics Statement

The study conformed to the Declaration of Helsinki and was approved by the Ethics Committee of Khon Kaen University (identifier number HE 561111).

## Author Contributions

BA-u: assessment, research fund application, ethical submission. NA: neurological examination, treatment. WP: MRI radiologist. TJ: assessment. OT: case collection, statistical analysis. WB: MRS technician. NN: case collection. KK: localization of voxel brain position. PA: study design, tDCS.

## Conflict of Interest Statement

The authors declare that the research was conducted in the absence of any commercial or financial relationships that could be construed as a potential conflict of interest.
